# Editorial: Multiomics advances: bridging gaps in understanding intricate biological systems

**DOI:** 10.3389/fpls.2026.1941133

**Published:** 2026-08-04

**Authors:** Vikram Singh

**Affiliations:** Department of Biotechnology, Amrapali University, Haldwani, Uttarakhand, India

**Keywords:** cytometry by time of flight (CyTOF), multi-omics, nanodroplet processing in one pot for trace samples (nanoPOTS), single cell proteomics by mass spectrometry (SCoPE), single-cell proteomics (SCP)

## Introduction

Single cell proteomics (SCP) is crucial for defining the cellular proteomics, cellular responses and their functions at a single cell level. SCP is a cutting-edge technology focuses on the study of proteins within individual cells, rather than a comprehensive analysis of proteins from the population of cells (Vistain and Tay, 2021). Since, biological systems are highly heterogeneous, consisting of intermixed subpopulations, different cell types and tissue substructures, hence, SCP enables a deeper understanding of cellular heterogeneity for studying complex biological systems (Mund et al., 2022). In addition, SCP identifies and characterizes rare cell types or cellular (sub) populations in an unbiased way which would remain unnoticed in classical bulk analyses. Absolute quantification of single-cell proteins and highly multiplexed protein measurements is possible with the recent technological advancements using innovative techniques such as, Single Cell ProtEomics by Mass Spectrometry (SCoPE, SCoPE2), Nanodroplet Processing in One Pot for Trace Samples (nanoPOTS), Flow Cytometry (FCM), Mass Cytometry (CyTOF) and Imaging Mass Cytometry (IMC) (Budnik et al., 2018; Specht et al., 2021; Zhu et al., 2018; Bandura et al., 2009; Robinson and Roederer, 2015). Recently, substantial development of nano-flow liquid chromatography mass spectrometry (nLC-MS) and advancement of data analysis algorithms allows identification and quantification of low abundant proteins in SCP (Ctortecka et al., 2023). Moreover, further expansion of SCP is being achieved with the development of new technologies including, miniaturized sample preparation, instrumentation advancements, optimization of chromatographic techniques, ultra-sensitive MS instrumentation, refined acquisition of mass spectra, and expansion of bioinformatics approaches (Kim et al., 2023; Nalla et al., 2025; Petrosius and Schoof, 2023).

## Technological advancement in the field of SCP

The first breakthrough in SCP was the development of an innovative method named, Single Cell ProtEomics by Mass Spectrometry (SCoPE-MS) (Budnik et al., 2018). SCoPE-MS allows the detection of thousands proteins in a single sample. SCoPE-MS was developed to investigate the proteome changes during stem cells differentiation into single embryo and embryonic stem cells to quantify over thousands of proteins in differentiating mouse embryonic stem (ES) cells. SCoPE-MS is broadly applicable for proteome profiling of single cell types with significantly better and faster identification than label-free methods. This method uses isobaric tandem mass tags (TMT) labeling to enable multiplex single-cell proteomics profiling and quantification of thousands of proteins in individual cells (Huang et al., 2007). Multiplexing is further improved by combining different cells types (single-cell, reference cell and carrier cells) using TMTpro to generate richer fragmentation spectra leading to significant increase in peptide and protein identifications (Budnik et al., 2018). The addition of carrier and reference cells increase the sample throughput, provides sufficient peptide ions and improves peptide identification in SCoPE-MS platform.

Further, SCoPE2 is designed to increase the sensitivity, accuracy, and throughput of single-cell protein analysis by optimizing the experimental conditions and data analysis protocols. SCoPE2 represents a significant advancement in SCP, enabling detailed and comprehensive study of cellular heterogeneity and protein function. Notably, SCoPE2 includes state-of-the-art methods including, minimal ProteOmic sample preparation (mPOP), and high-throughput automation for measurement of over 3000 proteins, as compared to the 1000 proteins identification by SCoPE (Specht et al., 2021; Petelski et al., 2021). Indeed, SCoPE2 offers several technological advancements over SCoPE in terms of improved sample preparation, minimize the sample loss, heterogenous samples analysis, multiplexing, and improved detection and quantification of low abundant proteins, with more accurate and reliable to increasing throughput and efficiency.

Later, an automated and miniaturized sample preparation method nanoPOTS was developed (Zhu et al., 2018). This cutting-edge microfluidic technology uses microwell plates to prepare single-cell samples for highly sensitive and precise protein analysis of very small biological samples. This chip-based nanodroplet platform uses micro-fabricated nanowell array chips designed to accommodate nanoliter volumes of sample for the analysis. Further, a nanoliter robotic pipetting system ensures minimal sample loss with high efficiency. In nanoPOTS, all the processes including, sample preparation, protein extraction, alkylation, and digestion are performed onto a nanowell-patterned single chip (Zhu et al., 2018). Further, ultrasensitive liquid chromatography coupled with ultra-sensitive tandem mass spectrometry (LC-MS/MS) provides comprehensive proteomic profiles (Cong et al., 2020). In addition, nested nanoPOTS (N2) chip was developed to quantify up to 1,500 proteins from 100 individual cells (Woo et al., 2021). The N2 chip is designed to accommodate 243 cells (total 27 nested nanowell, each contain 9 nanowells) on a single chip to achieve ten-fold sample processing and protein identification and quantification than nanoPOTS. Both, nanoPOTS and nanoPOTS2 offer several advantages including, miniaturized sample preparation in nanowells to minimizes sample loss, suitable for large-scale high-throughput analysis, and offer high sensitivity for the identification and quantification of thousands the proteins from very small samples.

Further, Antibody based SCP methods, such as Flow Cytometry (FCM), Mass Cytometry (CyTOF) and Imaging Mass Cytometry (IMC) are also uses for targeted protein detection and quantification in single cells. Cytometry-based single-cell flow cytometry (FCM) is a powerful technique used in SCP to analyze and sort individual cells based on their protein expression profiles. FCM based SCP (FCM-SCP) is a high throughput method, enable analysis of thousands of cells per second, using quantification of fluorescence emissions by fluorescent antibodies bound to single cells for large-scale studies (Robinson and Roederer, 2015; Lohani et al., 2023). FCM-SCP allows cell sorting by fluorescence-activated cell sorting (FACS) to achieve fluorescently-tagged antibodies for multiplexed sample analysis. In addition, it offers single cell resolution for detailed analysis of cellular heterogeneity within complex tissues. To overcome the limitations of requirement of large sample volume, a imaging flow cytometry (IFC) microsystem was developed. IFC comprises integrating of FCM and high-resolution imaging microscopy to enable high throughput analysis of SCP with better spatial resolution (Stavrakis et al., 2019; Miura et al., 2018). Likewise, multi-parametric IFC encompassing multi-color fluorescence and bright-field analysis has been developed to enable high resolution analysis (60,000cells/s) of cellular proteins with high sensitivity. This method enables precise sub-cellular analysis and high throughput localization of proteins (Holzner et al., 2021).

Later, a hybrid technology termed as mass cytometry/cytometry by time of flight (CyTOF) was developed by combining traditional FCM and MS. CyTOF is build to achieve highly multiplexed SCP analysis to explore the unparalleled insights of complex biological systems (Bandura et al., 2009). CyTOF provides precise quantitative measurement of protein at the single-cell level, revealing cellular heterogeneity. CyTOF is based on the concept of using heavy-metal isotopes to label antibodies rather than fluorescent tags to label the target proteins to avoid spectral overlap for simultaneous measurement of several proteins. CyTOF is also employed to identify the key cell surface markers and myogenic transformation factors to resolve the heterogeneity of myogenic compartments and myogenic progressions in skeletal muscle cells (Porpiglia et al., 2017). Importantly, CyTOF has been fabricated to study the changes in immune reconstitution at different phases of human cytomegalovirus reactivation after allogeneic hematopoietic stem cell transplantation (Stern et al., 2018).

Furthermore, imaging mass cytometry (IMC) was developed by combining mass cytometry with spatial imaging (Chang et al., 2017). IMC is a cutting-edge technique permits precise quantification, visualization and distribution of proteins within tissues providing comprehensive understanding of cellular interactions and microenvironments. Single cell IMC provides spatially-resolved analysis to enable the simultaneous detection of a broader range of protein markers for investigating phenotypic heterogeneity and tumor architecture. Notably, a 3D IMC was developed with high spatial resolution to examine the single-cell phenotype of cancer tissues and tumor invasion (Kuett et al., 2022). Further, the integration of IMC with high definition single cell analysis (HD-SCA) assays has been used to characterize rare tumor cells (Gerdtsson et al., 2018). In addition, using HD-SCA, an in-depth phenotyping of a liquid biopsy has been carried out for subcellular localization of markers within circulating tumor cells (CTCs).

## SCP integration with other omics approaches

The integration of SCP with other omics technologies such as single cell genomics (SCG), single cell RNA sequencing (scRNA-seq), single cell metabolomics (SCM) and single cell epigenomics (SCEpi) is an exciting frontier in systems biology. Combining single cell multi-omics provide comprehensive understanding of gene regulation and cellular behavior for understanding complex biological systems (Flynn et al., 2023). Integration of multi-omics approach reveals better insights into cellular processes, post-translational modifications, cellular activity and biological mechanism as compared to the single omics approach (Lee et al., 2020). Integration of SCP and scRNA-seq provides an important insight on correlated gene expression, post-transcriptional regulation, protein degradation, and translation efficiency (Jha et al., 2022). Moreover, Integration of SCP and SCEpi offers understanding on changes in the chromatin landscape influencing protein expression and cellular behavior at single-cell level (Vandereyken et al., 2023; Bi and Weng, 2024). Integration of SCP and SCM offers the exploration of metabolic flux in real-time, especially in response to environmental or therapeutic stimuli to provide deeper insights into the metabolic reprogramming (Orsburn, 2023). Cellular Indexing of Transcriptomes and Epitopes (CITE-seq) is a cutting-edge technique combines single-cell RNA sequencing (scRNA-seq) with antibody-based proteomic profiling. CITE-seq allows the measurement of both transcriptome (gene expression) and surface protein markers on the same individual cell to provide comprehensive understanding of cellular heterogeneity (Stoeckius et al., 2017). Recently, nanoSPLITS integrate proteomics workflow with well-established single-cell RNA sequencing technologies has enabled bi-modal analysis (Fulcher et al., 2024).

## Technological advancements and future directions

Technological advancements in SCP analysis is achieved with the development of microfluidics, enabling isolation and manipulation of individual cells in a controlled environment. Microfluidics and automation improve sample preparation by reducing sample loss and increasing high throughput analysis. Moreover, advancements in MS with the development of nLC-MS/MS, allows improved proteins profiling at single-cell level (Zheng et al., 2023). Furthermore, with the development of innovative antibody-based methods like single-cell antibody-based assays (CyTOF or FCM) and proximity extension assays (PEA) highly sensitivity protein detection at the single-cell level is achieved. In addition, development of new sample preparation techniques, such as nanoPOTS and proteoCHIP further improves the efficiency and accuracy of protein analysis at the single-cell level (Hartlmayr et al., 2021). CyTOF is one of the most widely used approach used for targeted SCP analysis, providing exploration of brain cell phenotype, immune cell differentiation, cell heterogeneity and cancer signaling (Zhang et al., 2020; Lun et al., 2019). Moreover, CyTOF in combination with cell imaging has been applied successfully to explore the subcellular distribution of proteins in individual cells during cellular processes, immune cell function and immune-therapeutic applications (Hiam-Galvez et al., 2021). Recently, single-cell CyTOF has been used to investigate heterogeneity in non-small cell lung cancer cell (NSCLC) lines (Neuperger et al., 2023). In addition, a single-cell barcode chip (SCBC) approach has been employed to explore cellular interactions and communications by measuring secretory proteins (Kravchenko-Balasha et al., 2016). SCBC analysis has emerged as a promising tool to study phenotypic markers, functional heterogeneity, signaling pathways, cell–cell interaction, cellular communication and immune cell response (Xue et al., 2017; Su et al., 2017). Single cell–resolution western blotting (scWB) system has been used to investigate the heterogeneity of circulating tumor cells (CTCs) in cancer patients (Sinkala et al., 2017). The scWB approach has also demonstrated its ability to interrogate the cellular heterogeneity and monitor single-cell differentiation of neural stem cells (Hughes et al., 2014). On the other hand, droplet microfluidics is used to detect surface receptors such as CD3 and CD19 markers from single-cell for cancers diagnosis, and measurement of receptor tyrosine kinase activity in lung cancer cells (Shahi et al., 2017; Ramji et al., 2014). Notably, surface-enhanced Raman spectroscopy (SERS) based microfluidic assay was used to monitor MCSP, MCAM, and LNGFR surface markers in CTCs (Reza et al., 2021). Meanwhile, CycMIST platform providing highly multiplexed profiling facilitate investigation of transcription factors, surface markers, and signaling proteins for Alzheimer’s disease (AD) (Yang et al., 2022). Meanwhile, nanoPOTS integrated with LCM have been used to differentiate the cell specific proteomes in human cancer tissues, rat brain, and mouse uterus (Piehowski et al., 2020). Particularly, the updated version of nanoPOTs, N2 has been used to identify cell-type-specific surface markers, to uncover cellular heterogeneity and cellular microenvironments (Woo et al., 2021). Recently, LCM-nanoPOTS has been modified with a hanging drop format to find tissue specific or cell-type specific biomarkers of mouse uterine tissues (Kwon et al., 2022). Indeed, single-cell chemical proteomics (SCCP) derived from SCoPE-MS has been developed to uncover heterogeneous responses of adenocarcinoma cells, drug resistance, and diverse cellular phenotypes upon drug treatments (Végvári et al., 2022). Moreover, iProChip–DIA demonstrated important drug targets, biomarkers and surface proteins associated with the NSCLC pathway from lung cancer cell (Gebreyesus et al., 2022). In addition, recent progress in SCP technologies has enabled the 3D mapping of proteome expression to explore single-cell heterogeneity, protein translocation events, and cell–cell interactions (Petrosius and Schoof, 2023).

These advancements offer crucial insight into the mechanisms of tumor metastasis, disease progression and treatment response, and accelerate the therapeutic applications of SCP. In addition, future prospects of SCP are driven by technological advancements including, robust development of bioinformatics and computational tools, and statistical methods, for interpreting the complex SCP data. Further advancements in SCP include automation, miniaturization of platforms, hybrid technologies, and integration with single cell omics data. Combining proteomics with imaging technologies like immune-histochemistry or super-resolution microscopy could provide detailed insights into cellular function. Additionally, the integration of artificial intelligence (AI) and machine learning (ML) can be helpful for the identification of complex proteomic data.

## Conclusion

SCP is a cutting-edge technology defining the cellular proteomics and their functions at a single cell level to enables a deeper understanding of cellular heterogeneity of complex biological systems. Absolute quantification of multiplexed protein measurements is achieved by the recent technological advancements. Innovative techniques such as, SCOPE, SCOPE2, nanoPOTS, CyTOF and IMC enables multiplexed proteome profiling of a complex biological system. Moreover, miniaturized sample preparation, instrumentation advancements, optimization of chromatographic techniques, ultra-sensitive MS instrumentation, refined acquisition of mass spectra, and expansion of bioinformatics approaches offers further analysis. Moreover, automation, miniaturization of platforms, development of hybrid technologies, robust development of bioinformatics and computational tools, and advanced statistical methods improves SCP. Integrated analysis of SCP with SCG, scRNA-seq, SCM and SCEpi develop a hybrid technology to provide comprehensive understanding of cellular behavior. Importantly, SCP provides valuable insights of tumor heterogeneity, disease mechanisms, therapeutic development, hematopoiesis, neurobiology, cellular development and developmental biology. Future prospects of SCP can be driven by integration of AI and ML to understand the complex proteomic data. In conclusion, SCP offers a unique perspective to deepen our understanding on molecular biology.
